# Suspended hybrid films assembled from thiol-capped gold nanoparticles

**DOI:** 10.1186/1556-276X-7-295

**Published:** 2012-06-06

**Authors:** Yu Xin Zhang, Ming Huang, Xiao Dong Hao, Meng Dong, Xin Lu Li, Jia Mu Huang

**Affiliations:** 1 College of Materials Science and Engineering, Chongqing University, Chongqing, 400045, People's Republic of China

**Keywords:** Nanoparticles, interfaces, thin films, hybrid, self-assembly, disassembly

## Abstract

In this work, we explored the formation processes of suspended hybrid thin films of thiol-capped Au nanoparticles (AuNPs) inside metal oxide tubular structures. We found that a balance between in-film interactions of the AuNPs and boundary interactions with metal oxides is a key in making these special organic–inorganic thin films. The hybrid films process many processing advantages and flexibilities, such as controllable film thickness, interfacial shape and inter-AuNPs distance, tuning of particle sizes, thiol population, chain lengths, and other new properties by introducing functional groups to thiol chains. Among their many unique features, the assembly-disassembly property may be useful for future on-off or store-release applications.

## Background

Ligands (e.g., alkanethiolate, phosphines, amines, peptides, DNA, and polymers) assisted organization of Au nanoparticles (AuNPs) has received great research attention over the past two decades [1-13]. As starting building units, surface-passivated AuNPs have been synthesized into various shapes and sizes [1-16]. Because of the presence of surface ligands, these AuNPs can self-assemble into larger constructs via van der Waals interactions existing among the ligand molecules. For example, superlattices, supported films, supracrystals, and sponges have been constructed with thiol-capped AuNPs [1,2,7,12-15]. In their two-dimensional arrays/assemblies, in particular, monolayer or multilayers of AuNPs have been prepared on solid supports (e.g., typically on polymeric films of transmission electron microscopy (TEM) copper grids) [1,2,7,12,13]. Although a significant research progress has been made, to the best of our knowledge, suspended hybrid films organized from thiol-capped AuNPs have not been reported. In view of their hybrid nature (i.e., organic thiols plus inorganic AuNPs), which can be finely tuned by chemical methods, the self-assembled thin films may provide desirable functionality and porosity for future applications.

In this work, as part of our recent effort in this area, we have prepared suspended hybrid thin films composed of thiol-capped AuNPs. Our findings reveal that inter-particulate interaction among the thiol-stabilized AuNPs plays an important role in film formation, while a certain degree of interaction with boundary materials is also essential in making these organic–inorganic thin films.

## Methods

In our synthesis, the AuNPs were synthesized according to a modified two-phase protocol [14-16], in which thiol molecules [1-dodecanethiol (DT) or 3-mercaptopropionic acid (MPA)] were used as surface capping agents for AuNPs (see supporting information (SI)-1 in Additional file 1). In a typical film preparation, anodic aluminum oxide (AAO) membrane templates were placed in a glass petri dish, and a drop of AuNP suspension (*ca* 0.02 mL; standard solution, see SI-1 in Additional file 1) was added onto each membrane, followed by a natural drying process. The same procedure was repeated several times in order to attain a desired film thickness. Afterwards, the AAO membranes were immersed in a plastic petri dish that contained an aqueous TiF_4_ solution (0.04 M) for a selected period of time (30 to 120 min). After the deposition of TiO_2_ nanotubes on their channels, the AAO membranes were washed with deionized water and air-dried at room temperature. The AAO templates were then removed by immersion in 1 M NaOH solution overnight. The solid products were collected and washed with deionized water and ethanol, followed by vacuum-drying. The product structure, morphology, and composition were investigated by TEM (JEM-2010, JEOL Ltd., Akishima, Tokyo, Japan), energy dispersive X-ray spectroscopy (EDX), and selected area electron diffraction.

## Results and discussion

**Figure 1:**
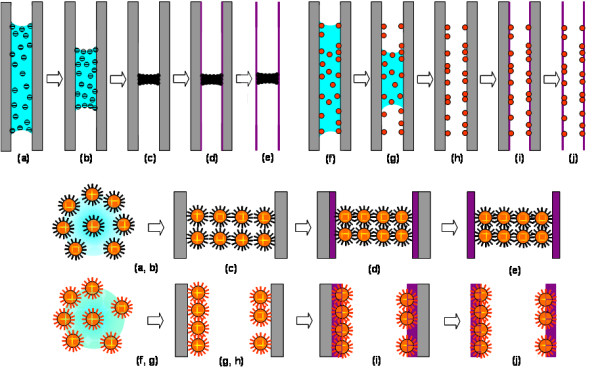
**Schematic illustrations.** (**a** to **e**) Formation of DT-capped AuNP hybrid films suspended inside TiO_2_ nanotubes and (**f** to **g**) formation of MPA-DT-capped AuNP-imbedded TiO_2_ nanotubes. Orange spheres represent AuNPs; black lines around AuNPs represent DT thiols; red lines around AuNPs represent MPA-DT thiols. Light blue backgrounds stand for a liquid phase (solvent: toluene or cyclohexane), grey vertical lines for AAO channels, and purple vertical lines for TiO_2_ nanotubes.

**Figure 2:**
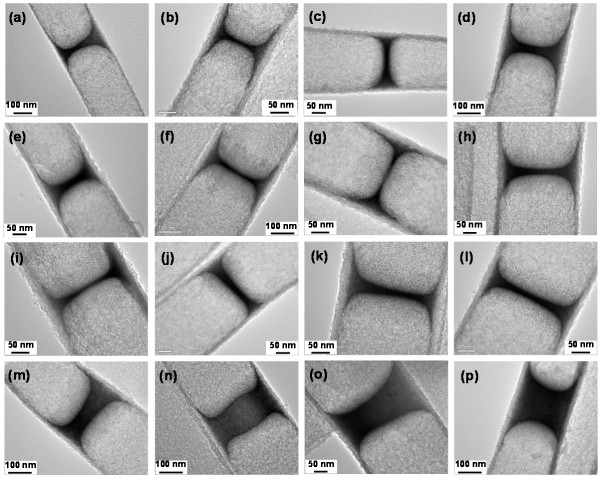
**TEM images.** (**a** to **p**) Concave hybrid films made from DT-capped AuNPs. See Additional file 1 (SI-2) for more information on these samples.

Figure 1 depicts two formation routes using two different types of functionalized AuNPs (see SI-1 in Additional file 1). The DT-capped AuNPs tend to agglomerate together via van der Waals forces generated among their surface alkyl headgroups, and they have a relatively weak interaction with AAO channels. Upon drying, the DT-capped AuNPs always stay in liquid phase (a, b) and form suspended films inside the AAO channels when the solvent (toluene or cyclohexane) is evaporated (c). Intriguingly, TiO_2_ nanotubes are grown selectively on the walls of the AAO channels, pushing the AuNP phase upwards (d). Alternatively, the TiO_2_ nanotubes can be grown firstly onto the AAO channels, and then, the AuNP films are introduced. Other sequential combinations are also viable. All these observations indicate that there is a stronger interaction among the DT-capped AuNPs than that between AuNPs and AAO or TiO_2_ walls. Figure 2 displays some representative TEM images of our suspended hybrid films assembled from DT-capped AuNPs (see SI-2 in Additional file 1). The film thickness can be controlled by the concentration of AuNPs and number of times that the AuNP solution is applied to the AAO membranes. On the other hand, the wall thickness of TiO_2_ nanotubes can also be manipulated by the concentration of TiF_4_ and hydrolysis time. As can be seen (see SI-3 in Additional file 1), the DT-capped AuNPs are located exclusively in the interiors of TiO_2_ nanotubes, and the smooth film surface reveals a concave shape of air-liquid (containing AuNPs) interface during the drying. Due to the hydrophobicity of the alkyl capping on the AuNPs, the shape of this interface was essentially maintained upon the TiO_2_ growth, AAO dissolution, and washing treatment, where polar solvents (e.g., mostly water) were used; it is further affirmed that the assembled films are completely bounded with circumferences of TiO_2_ nanotubes (see SI-4 in Additional file 1). The composition of the prepared products was also investigated with EDX (see SI-5 in Additional file 1), which confirms the formation process illustrated in Figure 1 phases a to e.

**Figure 3:**
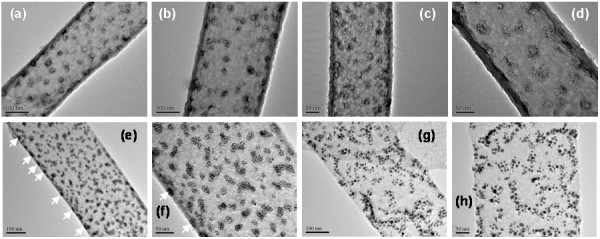
**TEM images.** TiO_2_ nanotubes imbedded with MPA-DT-capped AuNPs: (**a** to **d**) thick-walled nanotubes; (**e**,**f**) thin-walled nanotubes; and (**g**,**h**) thin-walled nanotubes after being heated at 250°C for 30 min. The imbedded AuNPs can be seen with the guide of white arrows.

When bifunctional thiol MPA is also introduced to the surface of AuNPs (see SI-1 in Additional file 1), the interaction between the AuNPs and AAO channel walls is significantly increased (Figure 1, phases f to i) since the carboxylate headgroups of MPA (see SI-6 in Additional file 1) are chemisorbed on Lewis acid sites of the AAO surface (Al^3+^ ions). In such a case, the MPA-DT-capped AuNPs can readily adsorb on the surface of AAO even when they are in a solution phase (Figure 1,phases f and g). During the growth of TiO_2_ nanotubes, the AuNPs are not relocated, and thus, they are incorporated (Figure 1, phases h to j) in the walls of TiO_2_ nanotubes, as seen in (Figure 3e,f). It is interesting to mention that these AuNPs aggregated into small islands (Figure 3; Figure 1, phases f and g) with a monolayer thickness, which indicates that there are weaker lateral interactions among the AuNPs through their long-chain DT molecules while MPA serves as a binder to the metal oxides [17]. Because they were immobile in the TiO_2_ matrix, the AuNPs did not grow much after the thermal treatment (Figure 3 g,h).

**Figure 4:**
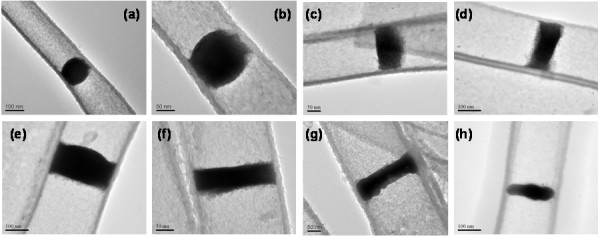
**TEM images.** Convex or flat hybrid films made from DT-capped AuNPs (with aid of MPA): (**a**,**b**) a spherical type film at different magnifications, (**c** to **f**) flat films, and flat films after being heated (**g**) at 150°C for 120 min and (**h**) at 250°C for 120 min.

Apart from the concave interfaces (H-shape, Figure 2), convex interfaces can also be attained. We find that when MPA is added to the existing DT-capped AuNPs, spherical aggregation takes place (see SI-7 in Additional file 1). In this work, convex or flat interfaces have been prepared with addition of MPA, as shown in Figure 4. It is believed that in this case, MPA serves primarily as a surfactant on the surface of AuNP aggregates. Since carboxylate groups are hydrophilic (see SI-6 in Additional file 1), the AuNPs become less soluble in toluene or cyclohexane and tend to form convex or flat surfaces (Figure 4).

Concerning their applications, we note that this type of hybrid films (or hybrid membranes) possesses many structural advantages and processing flexibilities: (1) AuNPs (or other metallic NPs) can be tuned with different sizes (e.g., monodisperse AuNPs, 2.1 ± 0.3 nm and 6.2 ± 0.6 nm; see SI-8 in Additional file 1); (2) inter-AuNP distance depends on the thiol population and chain lengths (e.g., 3.8 ± 0.4 nm and 8.6 ± 0.5 nm; see SI-8 in Additional file 1); (3) film thickness and interfacial shape can be engineered; and (4) new properties can be obtained by introducing functional groups to thiol chains. All these control factors will determine the final porosity and functionality of the suspended hybrid films in future applications (e.g., gas separation). For instance, these films can be used as a catalyst or a sealant for the enclosure of nano test tubes [18-20]. At a relatively low temperature, the AuNPs in these films are readily fused into single metallic gold plugs at 150°C to 250°C, as reported in Figure 4 g,h. On the other hand, while they are very stable in aqueous solution, the suspended hybrid films of AuNPs are soluble (and thus removable) in common organic solvents such as toluene and cyclohexane. The assembly-disassembly property that these films possess may be useful for on-off or store-release operations.

## Conclusions

In summary, we have explored the formation processes of suspended hybrid thin films of thiol-capped AuNPs inside metal oxide tubular structures. It has been found that a balance between the in-film interactions of the AuNPs and boundary interactions with metal oxides is a key in making these organic–inorganic thin films. Among many unique features, the assembly-disassembly property may be useful for on-off or store-release applications.

## Abbreviations

AAO, Anodic aluminum oxide; AuNPs, Au nanoparticles; DT, 1-dodecanethiol; EDX, Energy dispersive X-ray spectroscopy; MPA, 3-mercaptopropionic acid; SI, Supporting information; TEM, Transmission electron microscopy.

## Competing interests

The authors declare that they have no competing interests.

## Authors’ contributions

ZYX synthesized and characterized the hybrid films and wrote the manuscript. HM prepared the gold nanoparticles and TiO_2_ nanotubes. HXD and DM conceived and designed the experiments. LXL and HJM coordinated the study. All authors read and approved the final manuscript.

## Supplementary Material

Additional file 1: **The file shows eight supporting informations (SI) as follows:** (1) synthetic conditions, (2) preparation details for Figure 2 of the main text, (3) DT-capped AuNP films formed inside TiO_2_ nanotubes (at large magnification), (4) cross-sectional top view on the DT/AuNP films inside TiO_2_ nanotubes, (5) EDX and results, (6) molecular structures of DT and MPA, (7) MPA-assisted agglomeration of DT-capped AuNPs, and (8) TEM images for representative AuNPs used in this work. (PDF 1207 kb) Click here for file
