# Supplementary material for: Suspended hybrid films assembled from thiol-capped gold nanoparticles
Source: Nanoscale Res Lett. 2012 Jun 6;7(1):295. doi: 10.1186/1556-276X-7-295 (PMC3422164; doi:10.1186/1556-276X-7-295)
Supplement: Additional file 1: — The file shows eight supporting informations (SI) as follows: (1) synthetic conditions, (2) preparation details for Figure 2 of the main text, (3) DT-capped AuNP films formed inside TiO2 nanotubes (at large magnification), (4) cross-sectional top view on the DT/AuNP films inside TiO2 nanotubes, (5) EDX and results, (6) molecular structures of DT and MPA, (7) MPA-assisted agglomeration of DT-capped AuNPs, and (8) TEM images for representative AuNPs used in this work. (PDF 1207 kb) [file 1556-276X-7-295-S1.pdf]

Suspended Hybrid Films Assembled from Thiol-capped Gold Nanoparticles

Zhang Yu Xin, Huang Ming, Hao Xiao Dong, Dong Meng, Li Xin Lu, Huang Jia Mu

College of Materials Science and Engineering, Chongqing University, Chongqing, 400045,P.R.China

Email: zhangyx_cqu@163.com

SI-1 Synthetic conditions

Preparation of "standard gold solution"

DT-capped AuNPs: Au nanoparticles (AuNPs) were prepared according to a modified Brust's two-phase protocol. Briefly, a hydrogen tetrachloroaurate trihydrate ($HAuCl_4{\cdot}3H_2O$, at 30 mM) aqueous solution (3.0 mL) was added to a tetractylammonium bromide (TOAB, at 50 mM) containing toluene solution (3.6 mL), and the resultant solution was thoroughly mixed, during which the aqueous phase turned from yellow to colorless while the organic phase turned orange as a result of the transformation of $[AuCl_4]^-$ with TOAB cations. Under the stirring condition at room temperature, the solution was further mixed with 1-dodecanethiol (DT, 0.11 M in toluene, e.g., 0.82 mL (Au/DT = 1)) for 15 min, followed by adding a freshly prepared sodium borohydride ($NaBH_4$, at 0.44 M in deionized water, 2.05 mL) solution. The above mixture immediately turned from orange to deep brown, and the resulting AuNPs suspension was continuously stirred for another 15 min. Afterwards, the resulting AuNPs suspension in toluene phase was divided into eight groups and they were washed with ethanol (3 mL for each group) respectively. The solid products were collected via centrifugation and dried at room temperature. In a typical experiment, each group mentioned above was dispersed into 2 mL toluene or cyclohexane again for the film making process. These AuNPs suspensions were denoted as "standard gold solutions". The particle size of AuNPs in these "standard gold solutions" was controlled by the molar ratio of Au/DT (from 0.02 to 10) adopted in synthesis or a post-synthesis heat-treatment at 150^oC (see SI-8). Therefore, different sizes of AuNPs could be tested for the film formation.

MPA-DT-capped AuNPs: A hydrogen tetrachloroaurate trihydrate ($HAuCl_4{\cdot}3H_2O$, at 30 mM) aqueous solution (3.0 mL) was added to a tetractylammonium bromide (TOAB, at 50 mM) containing toluene solution (3.6 mL), and the resultant solution was thoroughly mixed, during which the aqueous phase turned from yellow to colorless while the organic phase turned orange as a result of the transformation of $[AuCl_4]^-$ with TOAB cations. Under the stirring condition at room temperature, the solution was further mixed with 1-dodecanethiol (DT, 0.11 M in toluene, 0.82 mL) and 3-mercaptopropionic acid (MPA, 0.11 M in toluene, 0.82 mL) for 15 min, followed by adding a freshly prepared sodium borohydride ($NaBH_4$, at 0.44 M in deionized water, 2.05 mL) solution. The above mixture immediately turned from orange to deep brown, and the resulting AuNPs suspension was continuously stirred for another 15 min. Other follow-up steps were the same as the above description.

Pretreatment of AAO membrane templates

Prior to deposition of TiO_2, as-received AAO membrane templates (Whatman, Anodisc 13, 0.2 μm membrane Discs) were ultrasonically cleaned in ethanol for about 5 min and dried at room temperature in laboratory air.

Preparation of hybrid films assembled from AuNPs

See the preparation in the main text.

SI-2 Preparation details for Figure 2 of main text

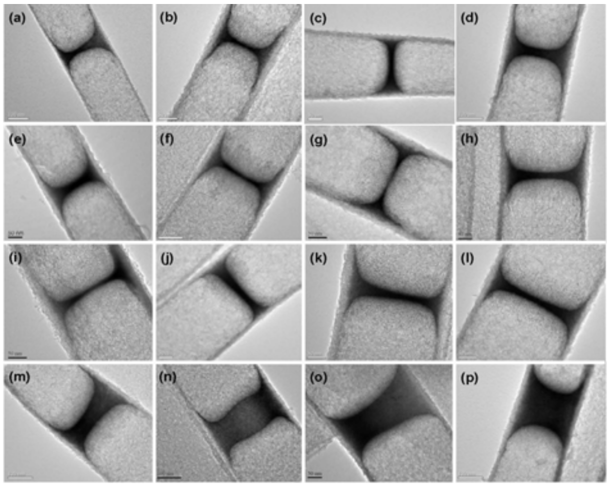

Three types of preparation sequences investigated (also see the main text):

AAO + AuNPs + TiO_2: (e) (Au/DT = 2);

AAO + AuNPs + TiO_2 + AuNPs: (o) (Au/DT = 1), (p) (Au/DT = 5); and

AAO + TiO_2 + calcination (400°C, 60 min) + AuNPs: (a), (b), (c), (d), (f), (g), (h), (i), (j), (k), (l), (m), (n) (Au/DT = 1).

Note: The different preparation sequences/treatments do not cause difference in the final products, which indicates that the interactions between the AuNPs and AAO channel walls (or TiO_2 nanotubes) are quite similar, and the final thickness of the hybrid films depends on the particle size and the amount of AuNPs added.

SI-3 DT-capped AuNPs films formed inside TiO_2 nanotubes (at large magnification)

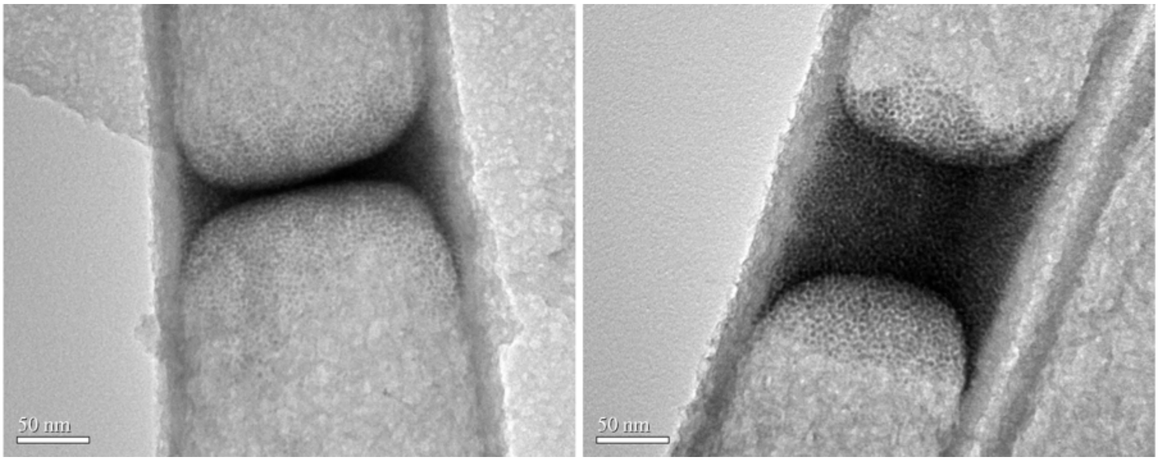

SI-4 Cross-sectional top view on the DT/AuNPs films inside TiO_2 nanotubes

Note: Short, broken TiO_2 nanotubes were deliberately selected, because they were able to stand vertically on the TEM copper grids.

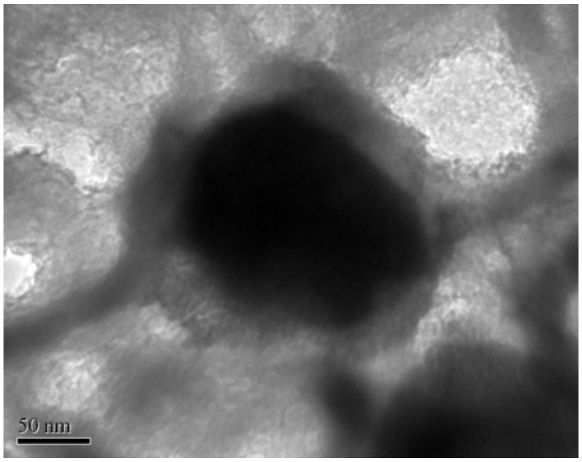

SI-5 EDX and results

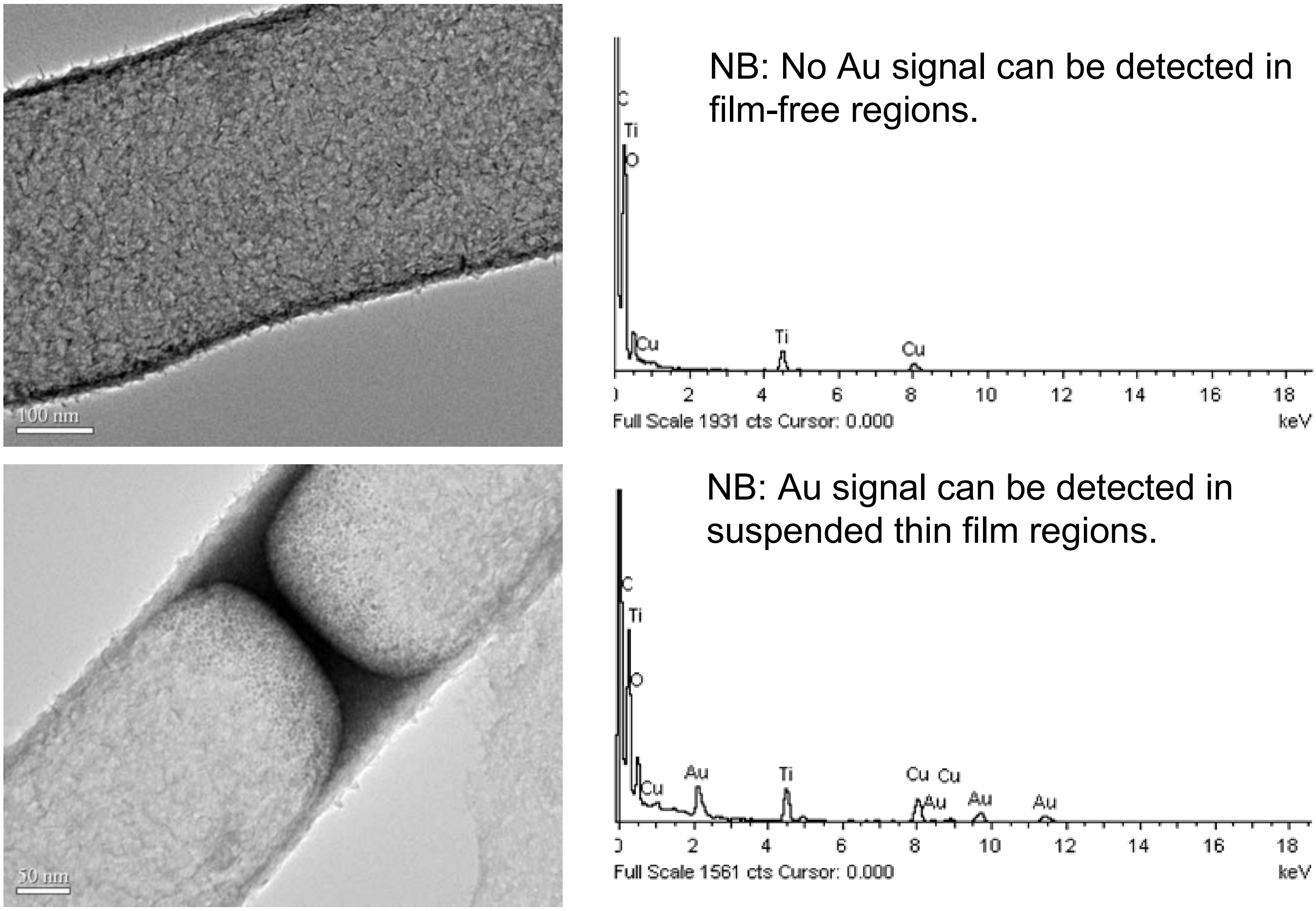

SI-6 Molecular structures of DT and MPA

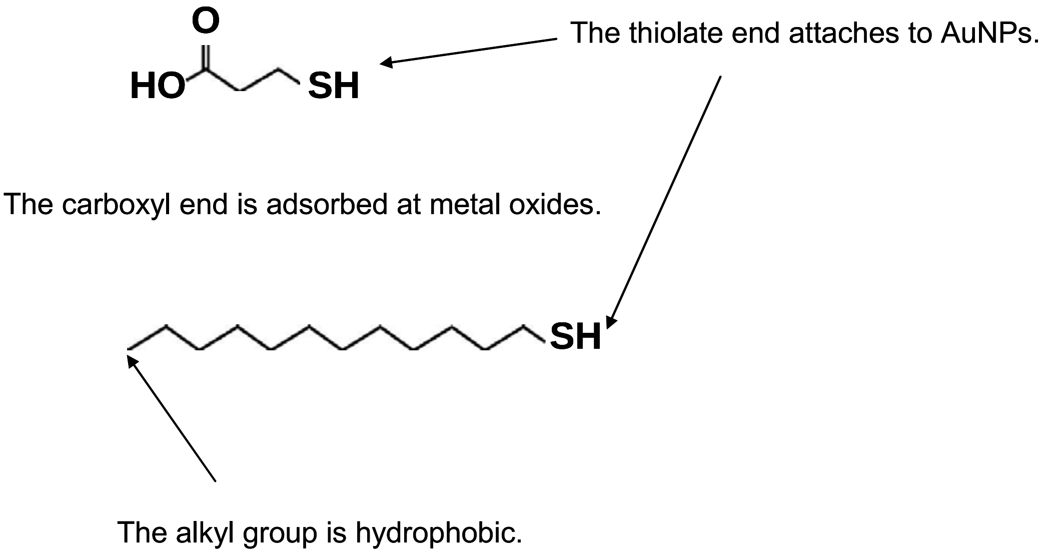

SI-7 MPA-assisted agglomeration of DT-capped AuNPs

0.2 mL pure 3-mercaptopropionic acid (MPA, Lancaster, 99%) was added to the suspension of DT-capped AuNPs (1 mL; Au/DT = 5 in cyclohexane; ethanol washed). Some precipitation (i.e., AuNPs aggregate) came out during the stirring. After the experiment, the solid products could not be dispersed in toluene.

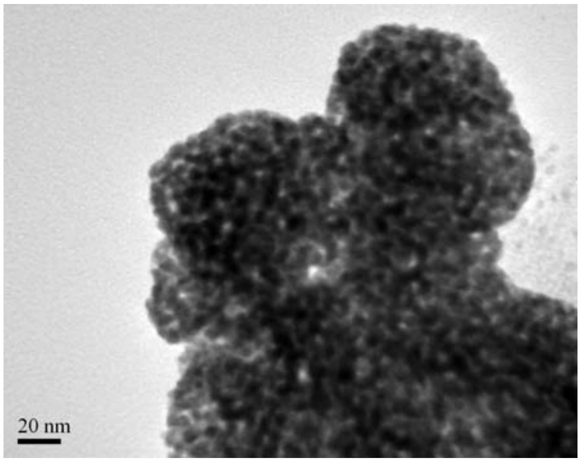

SI-8 TEM images for representative AuNPs used in this work

As prepared AuNPs (Au/DDT = 2):

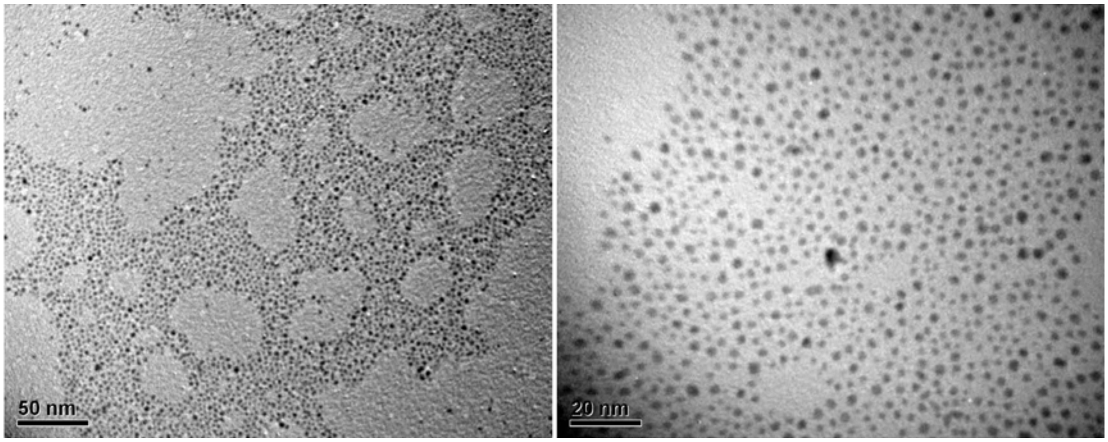

Particle size = 2.1 ± 0.3 nm

Inter-particle distance (center to center) = 3.8 ± 0.4 nm

Inter-particle space = 3.8 nm – 2.1 nm = 1.7 nm

The above AuNPs (Au/DDT = 2) were dried and heated at 150°C for 30 min:

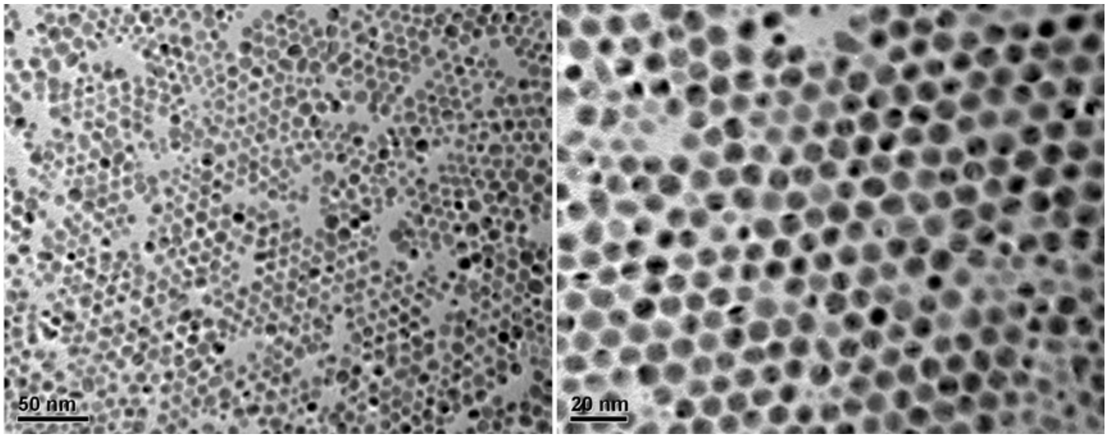

Particles size = 6.2 ± 0.6 nm

Inter-particle distance (center to center) = 8.6 ± 0.5 nm

Inter-particle space = 8.6 nm – 6.2 nm = 2.4 nm
